# Remarks on the Best Means of Securing Good Teeth

**Published:** 1858-09

**Authors:** J. Richardson


### REMARKS ON THE BEST MEANS OF SECURING GOOD TEETH.

(READ BEFORE THE "AMERICAN DENTAL CONVENTION," CINCINNATI.)

BY J. RICHARDSON.

MR. PRESIDENT AND GENTLEMEN :

THE question now before the convention, relating as it does to the best means of *securing* good teeth, may be justly regarded as a very important one. I use the term in no unmeaning sense. No problem in medical science whose solution affects the public health and welfare, can be of much greater moment than the one now under consideration.

Bad teeth in the aggregate, constitute a public calamity which it well becomes us by all possible means to mitigate if not prevent. The question is an important one, too, because, I believe, its earnest discussion will open up new subjects of thought and inquiry into the essential nature of some of the diseases of the teeth about which the profession has not heretofore sufficiently concerned itself. It is a question that carries us fairly back to *first causes.* Without some rational and well-grounded views of the agencies or influences, proximate and remote, concerned in the production of the ordinary diseases of the teeth, we can not hope to make much progress in the application of the means best calculated to *exempt* them from disease. All prophylactic measures are wisely and necessarily founded upon this requirement; and it loses none of its force in its application to the present question.

I propose, therefore, to inquire as briefly as possible into what I concieve to be the *essential nature* or *ultimate origin* of dental caries, and then to consider some of the indications that seem naturally to follow. I have mentioned caries because it is an affection that overshadows all others in importance and frequency.

The chemical theory of decay, as far as it applies in explanation of the active and immediate agencies concerned in its production, we believe to be correct. All concur in the belief that caries in its immediate development proceeds from causes external to the teeth. As a theory in explanation of its *proximate* cause it is sanctioned by the whole profession, and it seems to us to be warranted by all the facts and phenomena of that disease. But though true in its current application to caries, it is still to my mind, an unsatisfactory theory, inasmuch as it does not furnish us a full and sufficient explanation of all the *conditions* of decay. Its tendency is to circumscribe our remedial appliances by furnishing us but a *partial* view of the etiology of the disease in question.

I am fully persuaded, sir, that dental caries in its remote or primary origin is essentially a *disease of nutrition*, characterised by defective organization of structure, and that the open form of decay is but the active development of an original defect made manifest to our senses by agents capable of acting upon a mal-organized tissue. I do not claim that this precedent condition of decay invariably exists. Causes greatly impairing the vitality of the teeth, conjoined with unusual virulence of chemical action, exposure of the dentinal structure by mechanical attrition, fractures of the enamel, and doubtless other causes, may concur to develop decay, but that caries, as it ordinarily presents itself, has primarily a nutritive or constitutional origin and that it is subsequently developed in its open, palpable form by accidental circumstances, we believe to be so constant a fact as to constitute a rule.

With this view of the origin of dental caries I believe that teeth perfectly organized at the time of their irruption from the gums, will, with proper subsequent attention to cleanliness, remain through an ordinary life time proof against the ordinary agencies usually concerned in their destruction.

That caries of the teeth has a constitutional origin we believe may be made to appear from the consideration of a single phenomenon that seems to be characteristic of this disease. I refer to its *symmeterical* character. So constant is this peculiarity that in detecting a decay of a tooth upon one side of the mouth, we turn almost instinctively to its mate of the opposite side, and we are seldom disappointed in finding one there in all respects similarly situated, either in its active or incipient stages. The teeth similarly located are not only alike decayed, but in very many instances the exact locality, configuration of cavity and physical characteristics of decay are imitated with almost daguerreotype exactness. It may be said that similar conditions exist equally favoring chemical action, and though this may be true as in proximate situations and in depressions upon the grinding surfaces of the teeth, yet it does not invalidate the force of the fact stated, or the inference to be drawn from it, for this symmetrical arrangement of decay occurs with equal uniformity in situations least of all others favorable to the action of the secretions, as upon the prominent and rounded portions of the teeth, sometimes upon the very point of a cusp ;—situations entirely free from extraneous accumulations and constantly washed by the fluids of the mouth and cleansed by the action of the lips and tongue. This arrangement is closely followed in all characters of decay, and in all possible locations and as a fact or phenomenon of caries within the observation of all, it becomes in my opinion, an important link in a chain of causes worthy of our careful consideration. To my apprehension the deduction is unavoidable that these points of decay were defectively organized either from perverted or interrupted nutrition during their formative stages. That normal dentine is oftentimes imperfectly organized has been clearly shown by Mr. Tomes, and when we reflect that the enamel pulp previous to and during ossification, is highly vascular, we can readily imagine how its proper and perfect organization may be interrupted by a multitude of causes effecting important morbid changes in the blood of both parent and child. It is an important fact in this connection that nutritive diseases and nutritive perversions are singularly prone to arrange themselves symmetrically. Paget in his excellent work on Pathological Anatomy, gives some curious illustrations of this tendency, and it is equally conspicuous in many skin diseases, in some forms of rheumatism and paralysis, in syphilitic ulcerations of the bones, etc.

If the view which I have hastily and but very imperfectly elaborated in explanation of the nutritive origin of caries is a correct one, I apprehend we shall have something to direct our attention to in the future besides discussing the relative potency of acids and alkalies in the production of caries. I would by no means detract from the importance of those means best calculated to *preserve* the teeth, but I would, in aid of these important means, seek to extend our vision beyond the merely chemical view of the subject. If, as I claim, the teeth acquire their *predisposition* to decay during their transition stages of development, the question now before us becomes exceedingly pertinent and interesting. What may be done to improve the nutrition of these organs; or in other words, what feasible means may be employed to secure to them the highest and most perfect development? Whatever is done must be done through the mother or the child.

The management of the mother during gestation should be so directed as to secure to the offspring a healthy organization. The means best calculated to secure this end, while they may be stated in general terms to be such as are in strict accordance with recognized laws of health, are too complex and diversified to admit of extended discussion in this place. The means are those that concern her moral, mental and physical well-being, and must be determined by the circumstances and necessities of each individual case. There is one feature, however, in the management of women during pregnancy and lactation to which I desire especially to call attention, and that relates to the administration of the phosphate of lime. Now I do not claim that in seeking to improve the condition of the teeth we shall ever be able to give to our means any special direction to these organs, for they are endowed with no distinct affinities, or any independent power of election not common to the general organism. The laws of nutrition during the healthy growth and development of the system are harmonious and uniform, and though the elements of the blood intended for the nutrition of the several tissues have their specific destination, it is in obedience to certain vital forces or affinities, and their final disposition is quite beyond our election. But if it can be made to appear that the phosphate of lime when administered to the mother during pregnancy and lactation does exercise a marked and controlling influence upon the general organism of the child, we will be waranted, I think, in assuming that it may be successfully employed as a means of securing a more complete and perfect development of the teeth. Whatever will give increased solidity and strength to the bony system, and greater vigor to the constitution generally, can not well fail to confer upon the teeth a more perfect organization.

I am aware that the efficacy of this treatment has been questioned and it is not long since we were told that as a remedy in rickets it has been tried and abandoned as utterly inefficient. That there is something more to be considered than the mere question of plus and minus in animal chemistry, and that a defective ingredient in any organ can not be supplied by pouring into the mouth its chemical equivalent.

That the treatment is an old one is true;—that it has been abandoned as utterly inefficient, is a statement not warranted by facts. Investigation and experiment prove that this is, in some degree at least, plainly a question of plus and minus, and that a defective ingredient in the proper nutrition of the tissues *may* be supplied by pouring into the mouth its chemical equivalent. What are the facts? I am indebted for some interesting data bearing upon this subject to a recent work on the diseases of women and children, by Prof. Bedford, of New York University. He quotes Guèrin, a French author, who he claims has shown that rickets are frequently *produced* in young infants in one of two ways. 1st. By not being nursed, but fed on improper food. 2d. By being nursed for too long a time, and confined exclusively to breast milk. He has succeeded in producing rickets in young animals, either by interrupting their regular course of nursing, or by confining them too long to the exclusive use of the mother's milk. The explanation of this is obvious. The mother's milk contains all the elements necessary for the perfect nutrition of the infant. One of these elements is the phosphate of lime, especially intended for the formation of bone. A child is taken from the mother immediately after birth, and put upon a diet, oftentimes, but illy suited to its feeble assimilative powers, and as often deficient in phosphate of lime, and thus rickets are induced by a deficiency of this important salt. On the other hand if the child is confined for too long a period, to the exclusive use of the mother's milk this secretion fails ultimately to yield a sufficient amount of this salt for the increasing wants of the expanding organism of the child and the same results follow, imperfect consolidation of the bones. I need not stop to make any application of this important fact to the practical duties and obligations of our calling. The evils resulting to the child from its premature withdrawal from its natural nourishment, as well as exclusive and protracted nursing beyond proper limits, is a subject coming legitimately within our scope as friends and counsellors to those over whom we may and should exercise a salutary control.

If rickets may be thus artificially produced in either of the ways mentioned, the question begins to look very like a question of *minus* in animal chemistry,—this power of arresting or interrupting the normal growth of the osseous system, by withholding a *sufficiency* of the earthy salts in the nutriment.

Again, on the other hand, Dr. Mouries, another eminent French physician, has shown that in rachitic children, the administration of the phosphate of lime not only improves nutrition, but will actually *arrest* the *progress* of the disease. Does not this involve a question of *plus* in animal chemistry, this supplying a defective ingredient of the system, by pouring into the mouth its chemical equivalent?

Dr. Mouries, upon the strength of his observations and experiments, makes the following significant statement: 1st, that the diseases and mortality of infancy are in great part due to the insufficiency of the phosphate of lime in the ordinary nourishment. 2d, that by mixing a certain amount of this salt with the daily food of nurses, pregnant women and children, both the number of deaths and diseases is greatly diminished. In confirmation of this opinion, he presents the following results: Of seventy children under one year of age, to which he administered phosphate of lime, the deaths were one in six while according to the official statistics, the deaths, under ordinary circumstances, in the city of Paris, within the first twelve months of existence, are one in four. The experience of Pegot Ogier, also confirms the views entertained by Dr. Mouries. He instituted the following experiment. He selected eighteen women, who in the aggregate had borne twenty-two children, eight of which had died in the first year, the fourteen others were weak and lymphatic. Under these unfavorable circumstances, the influence of proper nourishment, was fully tested. To some he gave food with the phosphate of lime, during their pregnancy, and to others during the period of lactation. During this change of diet they had eighteen children, only three of which died during the first year, from accidental causes, while the remaining fifteen were remarkable for good health. So that the same women, who with their ordinary nourishment, had lost *eight* children out of *twenty-two*, lost only *three* out of *eighteen*, when their diet was changed by the addition of phosphate of lime during pregnancy or lactation; and again, the former children were lymphatic and delicate, while the latter exhibited all the appearances of robust health. Dr. Beneke, an author quoted by Prof. Bedford, has also developed at some length, the efficacy of the phosphate of lime, not only in rickets, but likewise in scrofula and other wasting diseases.

It is proper to state here, that the results of many of these experiments were submitted to, and sanctioned by the French Academy of Medicine, so that they have not only received the quasi-endorsement of one of the most practical minds of our own country, (Prof. Bedford) but the unqualified sanction of the highest medical authority in France.

That the phosphate of lime, when administered internally, is capable of exerting prominent influences for good upon the organism and health, is, upon credible authority, sustained by statistical facts that must outweigh all manner of speculative reasoning upon the subject. It is upon this ground that I claim it may be used in a large class of cases with decided advantage, not that it will fix itself directly upon the teeth, inducing any very considerable increase in the deposit of ossific matter, but that it will, and can be made to prove advantageous by promoting an equable and harmonious action in the functions, not only of the general system, but in the dental pulp during all its stages of transition from its earliest development to its final transformation into tooth-substance proper. More than this can probably not be claimed for it, that much, I think may, in the light of present evidence, be accomplished by its administration.

